# Supplementary material for: Incidence and predictors of mortality among patients with head injury admitted to Hawassa University Comprehensive Specialized Hospital, Southern Ethiopia: A retrospective follow-up study
Source: PLoS One. 2021 Aug 19;16(8):e0254245. doi: 10.1371/journal.pone.0254245 (PMC8376017; doi:10.1371/journal.pone.0254245)
Supplement: Attachment — Submitted filename: Authors response to editor and reviewer comments.docx [file pone.0254245.s002.docx]

**Author’s response to Editor and Reviewers comments**

Additional Editor Comments (if provided):
This is a unique article describing the incidence and predictors of mortality among patients with head injury admitted to Hawassa University Comprehensive Specialized Hospital, Southern Ethiopia.

Response: Thank you for your inspiring comment.

- The manuscript is not written in Standard English

**Response**: We have revised the original submission to improve its text size and English language. The changes across the whole document have been highlighted in the “Revised Manuscript with Track Changes” file.

**Reviewers' comments:**

**Reviewer #1:**

- Dear authors, I read your work with great interest. At present, TBI is one of the most important topics when dealing with trauma patients all around the world.
  **Response**: Thank you for your positive outlook towards our work.
- The allover quality of your manuscript would benefit greatly from proofreading by an English native speaker

**Response**: We have made improvements to the language in the revised document.

- When using the term "polytrauma" you do not give the definition used at your institution to classify patients as polytraumatized. Since the definition in use can have a relevant influence on the prognosis of the treated patients you should clarify this issue.

**Response**: Patients who had suffered multiple trauma did not meet the eligibility criteria because the majority of them was could not survive for the first 24 hours and those patients for whom the data could not be obtained from reviewing the case histories were excluded from the study. This may introduce misclassification and bias the estimate. Therefore, we have reported only descriptive statistics.

- You do not mention the time from trauma to the start of treatment of your patients nor do you give the amount of time they suffered from conditions like hypoxemia, hypothermia, etc. - this would be of great importance to put your findings into perspective.
- **Response**: The study setting is the only neurosurgery reference center in southern Ethiopia. Thus, the patients in the study came across more than 100 kilometers and spent a lot of time before reaching the hospital. Therefore, in many cases, it was difficult to obtain the necessary information on pre-hospital care and the time elapsed between trauma and receiving care. Also, two-thirds of patients have had mild TBI, so they cannot seek urgent care. Similarly, measuring for how long the patients exposed to hypoxia and hypotension were not possible. Hence, we have used admission blood pressure and oxygen saturation as a baseline.
- Subgroups would greatly improve the relevance of your manuscript.

**Response**: Thank you for your meticulous comment. We have added it according to your suggestion and it also indicated in Table 2

- "(Error! Reference source not found.)". Please clarify!

**Response**: Thank you for your remedial remarks. We have corrected it.

- **Reviewer #2:**

Dear Authors, doing research to improve patients' treatment and outcomes especially in the presence of limited resources is very impressive and you have collected a lot of important data on your patients

Response: Thank you for your inspiring remarks.

- The author needs to consider the formatting guidelines of PLOS one.

**Response**: We have formatted the document so that it can conform to the Plos one manuscript formatting style.

- Why did you limit the patient sample to 1220 if you had more patients?
- **Response**: We have used appropriate sample size formula for cohort study this yields a 1220 sample size for our study. The sample size was statistically efficient and precise.
- Data on follow-up should be added
- Response: We have followed the treatment outcomes of patients and it was presented in graphs and tables.

Secondary injury being a major contributor of bad outcomes has long been known.

**Response**: the aim of health research was to proven or disprove existing evidence. Our study finding supports existing evidence, that will help to improve the treatment outcome of the patients.
